# On the Effects of Abstinence on the Approach of Acute Diseases; Abstracted from the Original Papers of Dr. E. Miller, of New York

**Published:** 1799-03

**Authors:** 


### *On the Effects of Abſtinence on the Approach of acute Diſeaſes; abſtracted from the original Papers of Dr.* E. MILLER, *of New York.*

THE following fact ſeems to deſerve more attention than it commonly obtains:—In a diſtrict of the United States, diſtinguiſhed for the prevalence of the epidemic diſeaſes of ſummer and autumn, it is often aſſerted by ſenſible fenfible and acute obfervers, that they are accuftomed to obviate the attack of fevers, apparently approaching, by *rigid abftinence* from food. This abftinence, begun as foon as they perceive the feelings of indifpofition, ufually known to be the fore-runners of fever, is continued till fuch feelings ceafe, till appetite be reftored, and generally, indeed, till the calls of hunger become importunate. On different occafions, this procefs is of various duration, fometimes twenty-four, thirty-fix, and even forty-eight hours, according to the nature and exigencies of the cafe. The fuccefs of this regimen is commended by fuch as have experienced it, in ftronger terms than would be proper here to repeat, and perhaps ftronger than the reality of the cafe can juftify.

Although the obfervation abovementioned comes, in the prefent inftance, from a popular fource, the effects of abftinence, in obviating the approach of acute difeafes, have not efcaped the notice of the moft eminent phyficians. In the writings of HIPPOCRATES, we perceive the ftrong impreffion he had received on the fubject. SYDENHAM is ftill more explicit. In his account of the continued fever of the years 1673, 1674, and 1675, confi-dered by Dr. CULLEN as a variety of fynocha, or inflammatory fever, it is afferted that he often cured this, as well as other kinds of fever, in the beginning, merely by directing diluents, and prohibiting every kind of aliment. Thus he relieved his children and intimate friends, (to ufe his own words) *by making them faft ftrictly for two or three days.* And, befides medical authority, we may alfo adduce, in favour of this mode of preventing difeafes, the recommendation and practice of many men of letters, who have adopted it with the greateft zeal. The fedentary lives of fuch perfons, diminifhing keennefs of appetite, and augmenting the burden of repletion, and their experience of higher intellectual power in a fomewhat diminifhed degree of bodily vigour, may perhaps account for their attachment to this remedy.

After having pointed out the great advantages of total abftinence, at the commencement of acute difeafes; explained the important functions of the ftomach in the animal fyftem; and detailed with precifion the particular ftates of the body, as well as the various cafes in which abftinence is attended with beneficial effects, the author concludes this valuable paper with the following pertinent and ingenious remarks:—

"If the art of preferving health and prolonging life chiefly confift in a frugal and fparing ufe of ftimuli, and adapting them with caution and fkill to the fluctuating circumftances of the vital principle, we fhall furely find ftill ftill ftronger motives to apply this doctrine at the approach, and in the treatment of difeafes, when noxious powers of fuch preternatural violence invade the body, baffle every remedy, and ftimulate it to death. The regulation of this vital principle, here denominated excitability, the prefervation of it when prefent, and its reftoration when deficient, the reftraint of excitement within the bounds of moderation, the prohibition of all wafteful and undermining exceffes, will probably hereafter, at fome more enlightened æra of medicine, form a fyftem of rules for the management of health, and the prevention of difeafe, for the enjoyment of fenfe, and the refinement of intellect, which, inftead of the prefent feverifh dream of human life, will prefent a confummation of improvement and happinefs, which we now afcribe to fuperior beings.

"I have thus undertaken to examine a noted popular obfervation, to inquire into its truth, and to demonftrate its confiftency with the moft eftablifhed principles of the animal œconomy. If I do not miftake, it has been proved, that abftinence will be often a complete, generally an ufeful, and almoft always *a fafe means of obviating the approach of acute difeafes.* And, in a word, if it were poffible to offer to mankind a maxim of univerfal application to the treatment of incipient fevers, in all their variations and circumftances, I fhould be inclined to hazard the following aphorifm: *When fymptoms, denoting the approach of acute difeafes, are difcovered, abftain, for a proper length of time, from all aliment.*"

